# Prevalence and correlates of medication non-adherence among kidney transplant recipients more than 6 months post-transplant: a cross-sectional study

**DOI:** 10.1186/1471-2369-14-261

**Published:** 2013-12-01

**Authors:** Francis L Weng, Sheenu Chandwani, Karen M Kurtyka, Christopher Zacker, Marie A Chisholm-Burns, Kitaw Demissie

**Affiliations:** 1 Saint Barnabas Medical Center, Renal & Pancreas Transplant Division, 94 Old Short Hills Road, East Wing, Suite 305, Livingston, New Jersey 07039, USA; 2 Department of Epidemiology, Rutgers School of Public Health, 683 Hoes Lane West, Piscataway, New Jersey 08854, USA; 3 Novartis Pharmaceuticals Corporation, One Health Plaza, East Hanover, New Jersey 07936, USA; 4 University of Tennessee Health Science Center, College of Pharmacy, 881 Madison Ave, Suite 264, Memphis, Tennessee 38163, USA

**Keywords:** Kidney transplantation, Epidemiology, Compliance, Adherence

## Abstract

**Background:**

Among kidney transplant recipients, non-adherence with immunosuppressive medications frequently precedes allograft loss. We sought to determine the prevalence and correlates of medication non-adherence among kidney transplant recipients.

**Methods:**

We performed a single-center, cross-sectional study of kidney transplant recipients who were at least 6 months post-transplant. We measured self-reported adherence using the Immunosuppressive Therapy Adherence Scale (ITAS, which is scored from 0 to 12, where higher scores indicate increased adherence) and barriers to adherence using the Immunosuppressive Therapy Barriers Scale (ITBS). We also used validated scales to measure perceived stress, health literacy, anxiety, depression, and interpersonal support.

**Results:**

The 252 patients included in the study were 59.9% male, 27.0% Black, and at a median of 2.9 years post-transplant (interquartile range [IQR] 1.4-5.8). On the ITAS, 59.1% scored a perfect 12, 26.6% scored 10–11, and 14.3% scored 0–9. In univariate models, non-adherence (defined as ITAS score ≤9) was significantly associated with increased scores on scales for perceived stress (OR 1.12, 95% CI 1.01-1.25) and depression (OR 1.14, 95% CI 1.02-1.28), and with more self-reported barriers to adherence on the ITBS (OR 1.15, 95% CI 1.08-1.22). After adjusting for sociodemographic factors, stress and depression were not associated with non-adherence. Higher scores on the ITBS (corresponding to more self-described barriers to adherence) were associated with lower scores on the ITAS (P < 0.001). Several individual barriers were associated with non-adherence.

**Conclusions:**

Among prevalent kidney transplant recipients, a minority is non-adherent. Practical barriers to adherence may serve as promising targets for future interventions.

## Background

Among recipients of kidney transplants, non-adherence with prescribed immunosuppressive medications commonly occurs and frequently precedes allograft loss [1-3]. A recent systematic review reported that in fifteen cross-sectional studies, a median of 22.3% of kidney transplant recipients were non-adherent [1]. Furthermore, in ten cohort studies, a median of 36.4% of kidney allograft losses were associated with prior non-adherence [1]. Compared to recipients of other solid organ transplants, kidney transplant recipients may demonstrate higher rates of non-adherence [4].

Several factors are associated with post-transplant medication non-adherence [5]. Patient-related factors associated with non-adherence include younger age [2,6,7], increased time since the transplant [6], and possibly Black race [8]. Possible barriers to adherence include patients’ personal schedules and routines [9], characteristics of the medicines and their dosage and schedules [8,9], practical issues related to access to medications and pharmacy refills [9], and medication costs [10]. Provider- and health systems-related factors also may contribute significantly to post-transplant medication adherence [4]. Unfortunately, few trials have tested interventions designed to increase adherence among transplant recipients [11]. Development of effective adherence interventions for transplant recipients requires a better understanding of factors potentially associated with non-adherence.

In this study, we sought to determine, among a population of stable, adult kidney transplant recipients who were more than 6 months post-transplant, (1) the prevalence of self-reported medication non-adherence, (2) psychosocial correlates of non-adherence, and (3) self-reported barriers to medication adherence.

## Methods

### Study design

A cross-sectional study was conducted in the outpatient transplant clinic of the Renal and Pancreas Transplant Division at Saint Barnabas Medical Center in Livingston, New Jersey, USA. To be eligible for this study, patients had to (1) have a functioning kidney-only transplant; (2) have received the transplant at Saint Barnabas Medical Center; (3) be at least 6 months post-transplant; (4) be 18 years of age or older at the time of the study; (5) be able to understand English; and (6) give informed consent. We excluded patients who had received a non-renal solid organ transplant, a simultaneous pancreas-kidney transplant, or more than one kidney transplant. The study was approved by the human subjects Institutional Review Boards at both Saint Barnabas Medical Center and the University of Medicine and Dentistry of New Jersey.

### Study procedures

We enrolled kidney transplant recipients who came for an appointment in our outpatient transplant clinic between May and September 2011. Prior to each clinic session, we identified the scheduled patients who met the study eligibility criteria. During the clinic visit, the nephrologist described the study to patients and invited them to participate. Interested patients then met with study personnel to confirm study eligibility, provide informed consent, and complete the study’s self-administered questionnaires. Study instruments were all completed in-person at the time of the clinic and study visit. After completing the questionnaires, study participants were given a $15 gift card in appreciation of their participation.

### Self-report instruments

Adherence was measured using the self-administered Immunosuppressive Therapy Adherence Scale (ITAS). The ITAS is a 4-item, 12-point scale that has been validated in kidney transplant recipients [12] and used in other studies of medication adherence [13,14]. A higher score corresponds with increased adherence.

Barriers to adherence were measured using the Immunosuppressive Therapy Barriers Scale (ITBS), a reliable and validated scale (Cronbach’s alpha 0.91) [15]. The ITBS is a 13-item scale consisting of 5-point Likert responses that rate self-reported agreement with eight “uncontrollable” factors and five “controllable” factors. Scores range from 13 to 65. A higher score corresponds with more barriers to adherence.

Participants also completed self-report symptom rating scale, including Hospital Anxiety and Depression Scale (HADS) [16], Interpersonal Support and Evaluation List-12 (ISEL-12) [17,18], and Perceived Stress Scale-4 (PSS-4) [19,20]. On the HADS, anxiety and depression are each measured on a 22-point scale from 0 to 21, with a higher score denoting more anxiety or depression. Scale subscores for anxiety and depression of 0–7, 8–10, and 11–21 corresponded with no, doubtful, or definite anxiety or depression, respectively [16]. On the ISEL-12, social support is scored from 12 to 48, with a higher score corresponding to higher social support. On the PSS-4, perceived stress is rated from 0 to 16, with a higher score correlating to increased perceived stress. There are no scores cut-offs for the ISEL-12 or PSS-4.

Participants also completed the Short Test of Functional Health Literacy in Adults (sTOFHLA) [21]. On the sTOFHLA, health literacy is measured from 0 to 36, with a score of 0–16 deemed as “inadequate”, 17–22 as “marginal”, and 23–36 as “adequate” literacy.

### Statistical analysis

Categorical variables were summarized as proportions, and their estimates across groups (e.g. adherence categories) were compared using chi-square testing or Fisher’s exact test as appropriate. Continuous variables that were not normally distributed were summarized as medians with 25%-75% interquartile ranges (IQRs) and compared using Wilcoxon rank-sum tests. We used binary logistic regression [22] to model the unadjusted associations between independent variables (psychosocial and demographic covariates) and self-reported non-adherence, with non-adherence defined as an ITAS score of 9 or less. The Likert responses on the ITBS were modeled as ordinal variables. Candidate variables with P < 0.20 in the univariate analysis were eligible for inclusion in the multivariate models [22]. For the multivariate models, we adjusted each candidate psychosocial variable for the demographic variables that were significant. We also examined the unadjusted associations of the individual ITBS items with non-adherence (defined by the ITAS). We did not include the ITBS scores or individual ITBS items in adjusted, multivariate models, given that these barriers to adherence are part of the causal pathway leading to non-adherence and do not function as confounders.

For continuous variables, linearity in the logit was confirmed. Goodness of fit of the multivariate logistic regression models was assessed using the Hosmer-Lemeshow test [22]. Two-sided P-values <0.05 were considered statistically significant.

## Results

### Study sample characteristics

From May through September 2011, we enrolled 252 kidney transplant recipients out of 603 screened (41.7%). At least 136 patients (22.6%) explicitly declined to participate, because of lack of interest or inability to complete the study instruments during their clinic visit. The remaining screened patients were not approached, due to clinical concerns (e.g. acute illness) at the time of the office visit. The characteristics of the study subjects are shown in Table 1. Participants had a median age of 54.7 years (IQR 44.6-62.9) and were a median of 2.9 years post-transplant (IQR 1.4-5.8). Over one-quarter (27.0%) were Black, 28.2% had an annual household income < $35,000 (for comparison, median household income in the United States was $50,054 in 2011 [23]), and 43.7% had private medical insurance. A high percentage of patients had a diagnosis of glomerular disease (40.5%) or received a kidney transplant from a live donor (62.7%). Median serum creatinine was 1.4 mg/dL (IQR 1.1-1.8).

**Table 1:** Socio-demographic and clinical characteristics of study population, based upon score on the Immunosuppressive Therapy Adherence Scale (ITAS)

**Characteristic**	**Overall**	**ITAS score ≤ 9**	**ITAS score = 10-11**	**ITAS score = 12**	***P***
	**(N = 252)**	**(N = 36)**	**(N = 67)**	**(N = 149)**	
Median age in years (IQR)	54.7 (44.6-62.9)	48.1 (38.1-61.1)	54.6 (37.7-64.3)	55.0 (46.3-62.6)	0.17
Median years since transplant (IQR)	2.9 (1.4-5.8)	3.3 (2.2-5.1)	2.7 (1.4-5.7)	2.6 (1.3-5.9)	0.47
Male, n (%)	151 (59.9%)	22 (61.1%)	36 (53.7%)	93 (62.4%)	0.48
Female, n (%)	101 (40.1%)	14 (38.9%)	31 (46.3%)	56 (37.6%)	
Race/ethnicity, n (%)					0.79
White	145 (57.5%)	20 (55.6%)	39 (58.2%)	86 (57.7%)	
Black	68 (27.0%)	12 (33.3%)	16 (23.9%)	40 (26.9%)	
Asian	14 (5.6%)	1 (2.8%)	6 (9.0%)	7 (4.7%)	
Hispanic	25 (9.9%)	3 (8.3%)	6 (9.0%)	16 (10.7%)	
Etiology of kidney disease, n (%)					0.57
Diabetes mellitus	42 (16.7%)	8 (22.2%)	11 (16.4%)	23 (15.4%)	
Hypertension	65 (25.8%)	13 (36.1%)	16 (23.9%)	36 (24.2%)	
Glomerulonephritis	102 (40.5%)	10 (27.8%)	27 (40.3%)	65 (43.6%)	
Other	43 (17.1%)	5 (13.9%)	13 (19.4%)	25 (16.8%)	
Highest education level, n (%)					0.65
11^th^ grade or below	16 (6.4%)	3 (8.3%)	4 (6.0%)	9 (6.0%)	
High school graduate or GED	50 (19.8%)	8 (22.2%)	9 (13.4%)	33 (22.2%)	
Some college	95 (37.7%)	14 (38.9%)	25 (37.3%)	56 (37.6%)	
College graduate or above	90 (35.7%)	11 (30.6%)	28 (41.8%)	51 (34.2%)	
Unknown	1 (0.4%)	0	1 (1.5%)	0	
Marital status, n (%)					0.72
Married	167 (66.3%)	22 (61.1%)	42 (62.7%)	103 (69.1%)	
Widowed, divorced, separated, or never married	78 (31.0%)	13 (36.1%)	22 (32.8%)	43 (28.9%)	
No response	7 (2.8%)	1 (2.8%)	3 (4.5%)	3 (2.0%)	
Annual household income, n (%)					0.51
0-$34,999	71 (28.2%)	15 (41.7%)	17 (25.4%)	39 (26.2%)	
$35,000-$74,999	57 (22.6%)	8 (22.2%)	17 (25.4%)	32 (21.5%)	
$75,000 and above	97 (38.5%)	9 (25.0%)	27 (40.3%)	61 (40.9%)	
Unknown	27 (10.7%)	4 (11.1%)	6 (9.0%)	17 (11.4%)	
Employment status, n (%)					0.05
Working full-time	92 (36.5%)	8 (22.2%)	27 (40.3%)	57 (38.3%)	
Working part-time	36 (14.3%)	4 (11.1%)	12 (17.9%)	20 (13.4%)	
Not working	124 (49.2%)	24 (66.7%)	28 (41.8%)	72 (48.3%)	
Primary health insurance, n (%)					0.62
Private insurance	110 (43.7%)	12 (33.3%)	34 (50.8%)	64 (43.0%)	
Medicare	106 (42.1%)	16 (44.4%)	24 (35.8%)	66 (44.3%)	
Medicaid	27 (10.7%)	7 (19.4%)	6 (9.0%)	14 (9.4%)	
Medicaid and medicare	7 (2.8%)	1 (2.8%)	2 (3.0%)	4 (2.7%)	
Charity or self-pay	2 (0.8%)	0	1 (1.5%)	1 (0.7%)	
Type of kidney transplant, n (%)					
Deceased donor transplant	94 (37.3%)	11 (30.6%)	22 (32.8%)	61 (40.9%)	0.35
Live donor transplant	158 (62.7%)	25 (69.4%)	45 (67.2%)	88 (59.1%)	
On dialysis prior to transplant, n (%)	190 (75.4%)	28 (77.8%)	51 (76.1%)	111 (74.5%)	0.84
Required dialysis during first post-transplant week, n (%)	30 (11.9%)	5 (13.9%)	4 (6.0%)	21 (14.1%)	0.22
Acute rejection episodes since transplant, n (%)	25 (9.9%)	8 (22.2%)	7 (10.5%)	10 (6.7%)	0.02
Calcineurin immunosuppressant, n (%)					
Cyclosporine	34 (13.5%)	6 (16.7%)	9 (13.4%)	19 (12.8%)	0.83
Tacrolimus	217 (86.1%)	30 (83.3%)	58 (86.6%)	129 *86.6%)	0.87
Median serum creatinine, in mg/dL (IQR)	1.4 (1.1-1.8)	1.59 (1.3-2.1)	1.35 (1.03-1.69)	1.38 (1.11-1.79)	0.06

*Abbreviation*: *IQR* Interquartile range.

*P-values* were obtained from chi-square test for proportions and Kruskal-Wallis test for medians.

### Self-reported adherence

**Figure 1:**
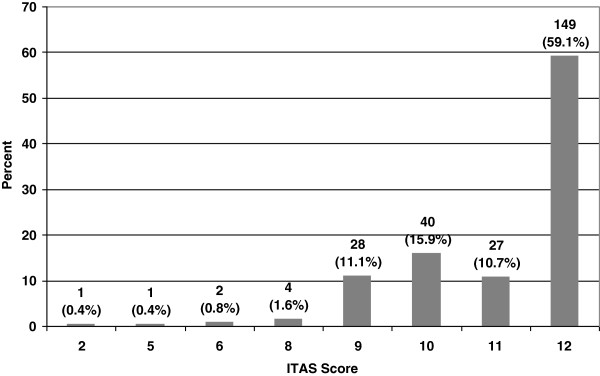
Distribution of scores on the Immunosuppressive Therapy Adherence Scale (ITAS).

The majority of study participants reported excellent adherence (Figure 1). 59.1% scored 12 out of 12, while 26.6% scored either a 10 or 11. The remaining 14.3% scored between 2 and 9 on the 12-point ITAS.

### Psychosocial measurements

The health literacy of our study population was high, with 97.6% having adequate health literacy on the sTOFHLA. The median sTOFHLA score was 35 (IQR 34–36). Only 6 patients (2.4%) had sTOFHLA scores of 22 or less, which suggest marginal or inadequate health literacy.

Most subjects reported lower anxiety and depression levels on the HADS. On the anxiety component of the HADS, 206 (81.8%) scored 0 to 7, 30 (11.9%) scored 8 to 10, and 15 (6.0%) scored 11 to 21, with 1 (0.4%) incomplete. The median anxiety score was 4 (IQR 2–7). On the depression component, 232 (92.1%) scored 0 to 7, 12 (4.8%) scored 8 to 10, and 5 (2.0%) scored 11 to 21, with 3 (1.2%) of responses incomplete and not scorable. The median depression score was 1 (IQR 1–3).

Interpersonal support was high, with a median ISEL-12 score of 45 (IQR 39–47). Perceived stress was variable, with a median PSS-4 score of 4 (IQR 1–7).

### Univariate and multivariate analysis of psychosocial factors associated with adherence

We performed unadjusted binary logistic regression models to examine the associations of candidate variables with non-adherence, defined as an ITAS score of 9 or less (Table 2). In the univariate logistic regression, non-adherence was associated with increased depression (on the HADS) and increased perceived stress (on the PSS-4) as well as lower household income and lack of employment. Younger age and increased anxiety appeared were not significantly associated with non-adherence but were considered candidate variables for the multivariate models (given their P < 0.20). Interpersonal support, health literacy, and clinical factors were not associated with non-adherence.

**Table 2:** Univariate and multivariate associations between Non-adherence (ITAS score of 9 or below) and psychosocial, socio-demographic, and clinical factors

**Predictor**	**Univariate models**			Adjusted, multivariate models*		
	**Odds ratio**	**95% confidence interval**	***P***	**Odds ratio**	**95% confidence interval**	***P***
**Psychosocial instruments**						
sTOFHLA Score	1.10	0.93-1.30	0.26			
HADS Anxiety Score	1.10	1.00-1.22	0.06	1.08	0.96-1.20	0.21
HADS Depression Score	1.14	1.02-1.28	0.02	1.13	1.00-1.28	0.06
ISEL-12 Score	0.99	0.94-1.05	0.71			
PSS-4 Score	1.12	1.01-1.25	0.04	1.10	0.97-1.24	0.12
ITBS Score	1.15	1.08-1.22	<.001			
**Socio-demographic factors**						
Age (years)	0.98	0.95-1.00	0.09			
Female (vs. male)	0.94	1.46-1.95	0.87			
Non-white race (vs. white)	1.10	0.54-2.24	0.79			
High school graduate and below (vs. some college and above)	1.28	0.59-2.77	0.53			
Not married or partnered (vs. married or partnered)	1.32	0.63-2.78	0.47			
Annual household income < $35,000 (vs. ≥ $35,000)	2.16	1.01-4.62	0.047			
Public health insurance (vs. private insurance)	1.66	0.79-3.49	0.18			
Unemployed (vs. employed)	2.32	1.10-4.88	0.03			
**Clinical factors**						
Living donor (vs. deceased donor)	1.42	0.66-3.03	0.37			
Primary diagnosis (vs. glomerulonephritis)						
Diabetes	2.17	0.79-5.94	0.13			
Hypertension	2.30	0.94-5.61	0.07			
Other	1.21	0.39-3.78	0.74			
Serum creatinine (mg/dL)	1.83	1.18-2.83	0.007			
Albumin (g/dL)	0.83	0.33-2.05	0.68			
6 months to 2 years since transplant (vs. >2 years since transplant)	0.53	0.26-1.09	0.08			

*Adjusted for socio-demographic factors that had P < 0.20 (age, income, employment).

In separate multivariate models, we adjusted each candidate psychosocial variable (anxiety, depression, and perceived stress) for candidate demographic variables with P < 0.20 in the unadjusted model (age, income, employment status). Therefore, we constructed three multivariable models. In these adjusted models, anxiety, depression, and perceived stress were no longer significantly associated with non-adherence (Table 2).

### Barriers to adherence

The median ITBS score was 16 (IQR 13–20). Higher scores on the ITBS (corresponding to more self-described barriers to adherence) were significantly associated with lower scores on the ITAS (P < 0.001).

Seven of the thirteen individual items in the ITBS were significantly associated with adherence on the ITAS (Tables 3 and 4). Patients with lower adherence on the ITAS were more likely to rate increased agreement with statements that they skip doses when they go out of town or when they feel depressed. Patients with lower adherence were more likely to rate increased agreement that they run out of medications, find it hard to remember to take their medications, miss doses due to perceived side effects, miss doses when out of their daily routine, and skip doses when short of money.

**Table 3:** Significant barriers to immunosuppressant adherence, based upon responses to the ITBS (Immunosuppressive Therapy Barriers Scale)

**ITBS questions**	**Overall (N = 252)**	**ITAS Score ≤ 9 (N = 36)**	**ITAS Score =10-11 (N = 67)**	**ITAS Score =12 (N = 149)**	**P-value**
	**N (%)**	**N (%)**	**N (%)**	**N (%)**	
**I skip doses of my immunosuppressant medication(s) when I go out of town**					0.04
Strongly disagree	214 (84.9)	24 (66.7)	61 (91.0)	129 (86.6)	
Disagree	26 (10.3)	7 (19.4)	5 (7.5)	14 (9.4)	
Neutral	5 (2.0)	2 (5.6)	0	3 (2.0)	
Agree	4 (1.6)	2 (5.6)	1 (1.5)	1 (0.7)	
Strongly agree	3 (1.2)	1 (2.8)	0	2 (1.3)	
**I miss doses of my immunosuppressant medication(s) when I feel depressed**					0.009
Strongly disagree	216 (85.7)	24 (66.7)	60(89.6)	132 (88.6)	
Disagree	27 (10.7)	7 (19.4)	7 (10.5)	13 (8.7)	
Neutral	4 (1.6)	2 (5.6)	0	2 (1.3)	
Agree	4 (1.6)	3 (8.3)	0	1 (0.7)	
Strongly agree	1 (0.4)	0	0	1 (0.7)	
**I often run out (or do not have enough) of immunosuppressant medication(s)**					<0.0001
Strongly disagree	202 (80.2)	21 (58.3)	48 (71.6)	133 (89.3)	
Disagree	30 (11.9)	5 (13.9)	12 (17.9)	13 (8.7)	
Neutral	11 (4.4)	6 (16.7)	3 (4.5)	2 (1.3)	
Agree	7 (2.8)	4 (11.1)	2 (3.0)	1 (0.7)	
Strongly agree	1 (0.4)	0	1 (1.5)	0	
Missing	1 (0.4)	0	1 (1.5)	0	
**It is hard for me to remember to take my immunosuppressant medication(s)**					<0.0001
Strongly disagree	209 (82.9)	24 (66.7)	50 (74.6)	135 (90.6)	
Disagree	35 (13.9)	8 (22.2)	16 (23.9)	11 (7.4)	
Neutral	5 (2.0)	2 (5.6)	0	3 (2.0)	
Agree	2 (0.8)	2 (5.6)	0	0	
Strongly agree	0	0	0	0	
Missing	1 (0.4)	0	1 (1.5)	0	
**I miss a dose of my immunosuppressant medication(s) when I think there may be side effects**					0.02
Strongly disagree	215 (85.3)	26 (72.2)	56 (83.6)	133 (89.3)	
Disagree	24 (9.5)	4 (11.1)	7 (10.5)	13 (8.7)	
Neutral	5 (2.0)	2 (5.6)	1 (1.5)	2 (1.3)	
Agree	7 (2.8)	4 (11.1)	2 (3.0)	1 (0.7)	
Strongly agree	0	0	0	0	
Missing	1 (0.4)	0	1 (1.5)	0	
**I miss doses of my immunosuppressant medication(s) when I get out of my daily routine**					<0.0001
Strongly disagree	173 (68.7)	113 (36.1)	31 (46.3)	129 (86.6)	
Disagree	39 (15.5)	9 (25.0)	13 (19.4)	17 (11.4)	
Neutral	8 (3.2)	3 (8.3)	4 (6.0)	1 (0.7)	
Agree	28 (11.1)	9 (25.0)	17 (25.4)	2 (1.3)	
Strongly agree	3 (1.2)	1 (2.8)	2 (3.0)	0	
Missing	1 (0.4)	1 (2.8)	0	0	
**I skip doses of my immunosuppressant medication(s) when I am short of money**					0.001
Strongly disagree	214 (84.9)	23 (63.9)	57 (85.1)	134 (89.9)	
Disagree	29 (11.5)	7 (19.4)	9 (13.4)	13 (8.7)	
Neutral	4 (1.6)	2 (5.6)	1 (1.5)	1 (0.7)	
Agree	3 (1.2)	2 (5.6)	0	1 (0.7)	
Strongly agree	2 (0.8)	2 (5.6)	0	0	

**Table 4:** Unadjusted, univariate model of ITBS items associated with non-adherence (ITAS score of 9 or below)

**Immunosuppressant Therapy Barrier Scale (ITBS) items**	**Odds ratio**	**95% confidence interval**	***P***
I have to take the immunosuppressant medication(s) too many times per day.	1.34	0.96-1.86	0.09
I have to take too many capsules (or tablets) of my immunosuppressant medication(s) at one time.	1.16	0.86-1.56	0.34
I cannot tell if my immunosuppressant medication(s) is (are) helping me.	1.18	0.86-1.62	0.30
I skip doses of my immunosuppressant medication(s) when I go out of town.	1.83	1.22-2.73	0.003
I miss doses of my immunosuppressant medication(s) when I feel depressed.	2.30	1.42-3.74	<0.001
I get confused about how to take my immunosuppressant medication(s).	1.14	0.39-3.36	0.81
I do not understand when to take my immunosuppressant medication(s).	0.73	0.26-2.08	0.55
I often run out (or do not have enough) of my immunosuppressant medication(s).	2.22	1.50-3.30	<0.001
It is hard for me to remember to take my immunosuppressant medication(s).	2.72	1.53-4.84	<0.001
I miss a dose of my immunosuppressant medication(s) when I think there may be side effects.	2.05	1.32-3.19	0.001
I sometimes skip doses of my immunosuppressant medication(s) when I feel good (or better).	1.68	0.71-3.97	0.24
I miss doses of my immunosuppressant medication(s) when I get out of my daily routine.	1.78	1.35-2.37	<0.001
I skip doses of my immunosuppressant medication(s) when I am short of money.	2.86	1.70-4.80	<0.001

Odds ratios reflects odds of higher degree of agreement with each statement, based upon responses on a 5-point ordinal Likert scale (Strongly agree to strongly disagree).

The remaining six items in the ITBS were not associated with adherence (Table 4). In particular, lack of knowledge about the benefits of transplant immunosuppression was not associated with adherence [as measured by the ITBS items “I get confused about how to take my immunosuppressant medication”; “I do not understand when to take my immunosuppressant medication(s)”; and “I sometimes skip doses of my immunosuppressant medication(s) when I feel good (or better)”].

## Discussion

In this cross-sectional study of prevalent adult recipients of kidney transplants, most patients were very adherent with their medications, at least by self-report. A minority of patients reported non-adherent medication-taking behaviors. Non-adherence was associated with higher perceived stress, anxiety, and depression. In adjusted, multivariate models, however, we could no longer detect any associations between non-adherence and stress, anxiety, and depression. The presence of self-described barriers to adherence was associated with non-adherence. These barriers to adherence may serve as potential targets for future interventions designed to increase medication adherence.

Our results confirm that a notable minority of kidney transplant recipients are non-adherent with their prescribed medications. Clearly, our study patients had been adherent enough to maintain a functioning allograft for a median of three years. Nevertheless, over 40% of patients were non-adherent in some form, as measured by the ITAS, and 14.3% were especially non-adherent, with an ITAS score of 9 or below. Other studies of prevalent kidney transplant recipients have reported similar rates of non-adherence [13]. Such long-term non-adherence may be associated with antibody-mediated rejection and allograft loss [3]. At least two ongoing clinical trials are testing interventions designed to increase adherence among prevalent kidney transplant recipients [24,25].

Our results also suggest possible targets for future interventions intended to increase medication adherence. Several specific barriers were significantly associated with non-adherence and may be amenable to modification. For example, changes in the patient’s routine, including travel, were associated with non-adherence; contingency plans may help patients remain adherent despite changes in daily routines [13]. Patients admitted to running out of their medications or simply forgetting to take them; reminder cues, systems, and alarms can prompt patients to take their medications and to refill their medication prescriptions. At least one ongoing trial is attempting to determine whether reminders can increase adherence among incident kidney transplant recipients [26]. Interventions that address these “practical” barriers to adherence may be effective in increasing adherence.

This study has several important limitations. First, we assessed medication adherence by self-report, using a single instrument (the ITAS). Other methods of measuring adherence include electronic monitoring [27-29], clinicians’ collateral reports [30,31], serum assays for immunosuppressive medication concentrations [32-34], pill counts, and prescription refill and claims records [35]. Use of multiple methods, rather than a single method such as self-report, may be the most valid way to detect and measure non-adherence [36].

Second, we categorized adherence using cut-off scores for the ITAS. Although the ITAS itself has been validated, these cut-offs and categories of ITAS scores have not been validated for their association with outcomes. The clinical significance of different ITAS scores and categories is uncertain. To our knowledge, the correlation between specific amounts of adherence (whether measured on the ITAS or other instruments) and post-transplant outcomes remains unknown.

Second, we measured adherence among transplant recipients at a single transplant center in the northeastern United States. Our results are not necessarily generalizable to other transplant centers. Transplant centers differ in their staffing levels, the frequency with which they follow-up transplant recipients, the cultural competency of their providers, and the instructions they give transplant recipients regarding medications. Provider-level and health systems-level factors that vary between transplant centers may affect patients’ adherence and contribute to inter-center variability in adherence [8].

Third, our convenience sample of prevalent transplant recipients who appeared for outpatient transplant follow-up was probably an especially adherent subset of kidney transplant recipients. Our study sample excluded patients who no longer have a functioning allograft or follow-up with our transplant center. To qualify for the study, patients had to appear for a scheduled clinic appointment, which is itself a marker for adherence. Only 41.7% of screened patients agreed to participate and complete our study questionnaires; we suspect that these study participants were more adherent than the overall population of screened patients. Our final study sample included large proportions of patients with favorable characteristics associated with increased allograft survival (e.g. recipients of live donor kidneys, patients with glomerular disease). Overall, this selection bias likely led us to overestimate the self-reported adherence of kidney transplant recipients in general.

Fourth, we were likely underpowered to detect associations between multiple factors and non-adherence. Although we examined over 250 transplant recipients, a study to examine the multiple factors plausibly associated with non-adherence may require a much larger study sample. Multi-center studies [6] may be necessary to accrue the larger numbers needed to properly study medication adherence.

Finally, we performed a cross-sectional study. This cross-sectional study design precluded meaningful analysis of the associations between adherence and acute rejection. For example, study participants with lower ITAS scores were more likely to have had prior rejection episodes. However, we lacked information on these patients’ adherence and ITAS scores prior to the rejection episodes. An alternative study design would be a prospective cohort study, in which transplant recipients are followed over time. A cohort study would permit correlation of medication adherence with subsequent transplant outcomes, such as rejection or renal function.

## Conclusions

In this single-center, cross-sectional study, a minority of recipients of kidney transplants was non-adherent with their medications. Non-adherence to medications was associated with increased anxiety, depression, and stress, but these associations did not persist in multivariate models. Practical barriers to adherence, such as forgetfulness and missing medications when one’s routine is different, were significantly associated with non-adherence. These practical barriers may serve as promising targets for future interventions to increase adherence among recipients of kidney transplants.

## Competing interests

This study was funded in part by Novartis Pharmaceuticals Corporation (East Hanover, New Jersey), as a research project with Drs. Weng and Demissie. Drs. Zacker is an employee of Novartis Pharmaceuticals. Dr. Chisholm-Burns receives licensing royalties from use of the ITAS and ITBS instruments from Pharmacotherapy Solutions.

## Authors’ contributions

FLW led the study conception and design; contributed to data acquisition, analysis, and interpretation; and drafted the manuscript. SC contributed to the study design and data acquisition, led the data analysis and interpretation, and contributed to revision of the manuscript. KMK contributed to the data analysis and interpretation and revision of the manuscript. CZ contributed to the study conception and design, data analysis and interpretation, and revision of the manuscript. MC-B contributed to the study design, data analysis and interpretation, and revision of the manuscript. KD contributed to the study conception and design, data analysis and interpretation, and revision of the manuscript. All authors read and approved the final manuscript.

## Pre-publication history

The pre-publication history for this paper can be accessed here:

http://www.biomedcentral.com/1471-2369/14/261/prepub

## References

[B1] Butler JA Roderick P Mullee M Mason JC Peveler RC Frequency and impact of nonadherence to immunosuppressants after renal transplantation: a systematic review Transplantation 2004 77 5 769 776 10.1097/01.TP.0000110408.83054.88 15021846

[B2] Denhaerynck K Dobbels F Cleemput I Desmyttere A Schafer-Keller P Schaub S De Geest S Prevalence, consequences, and determinants of nonadherence in adult renal transplant patients: a literature review Transpl Int 2005 18 10 1121 1133 10.1111/j.1432-2277.2005.00176.x 16162098

[B3] Sellares J de Freitas DG Mengel M Reeve J Einecke G Sis B Hidalgo LG Famulski K Matas A Halloran PF Understanding the causes of kidney transplant failure: the dominant role of antibody-mediated rejection and nonadherence Am J Transplant 2012 12 2 388 399 10.1111/j.1600-6143.2011.03840.x 22081892

[B4] Dew MA DiMartini AF De Vito Dabbs A Myaskovsky L Steel J Unruh M Switzer GE Zomak R Kormos RL Greenhouse JB Rates and risk factors for nonadherence to the medical regimen after adult solid organ transplantation Transplantation 2007 83 7 858 873 10.1097/01.tp.0000258599.65257.a6 17460556

[B5] Prendergast MB Gaston RS Optimizing medication adherence: an ongoing opportunity to improve outcomes after kidney transplantation Clin J Am Soc Nephrol 2010 5 7 1305 1311 10.2215/CJN.07241009 20448067 PMC2893061

[B6] Greenstein S Siegal B Compliance and noncompliance in patients with a functioning renal transplant: a multicenter study Transplantation 1998 66 12 1718 1726 10.1097/00007890-199812270-00026 9884266

[B7] Pinsky BW Takemoto SK Lentine KL Burroughs TE Schnitzler MA Salvalaggio PR Transplant outcomes and economic costs associated with patient noncompliance to immunosuppression Am J Transplant 2009 9 11 2597 2606 10.1111/j.1600-6143.2009.02798.x 19843035

[B8] Weng FL Israni AK Joffe MM Hoy T Gaughan CA Newman M Abrams JD Kamoun M Rosas SE Mange KC Race and electronically measured adherence to immunosuppressive medications after deceased donor renal transplantation J Am Soc Nephrol 2005 16 6 1839 1848 10.1681/ASN.2004121059 15800121

[B9] Gordon EJ Gallant M Sehgal AR Conti D Siminoff LA Medication-taking among adult renal transplant recipients: barriers and strategies Transpl Int 2009 22 5 534 545 10.1111/j.1432-2277.2008.00827.x 19175560 PMC3540791

[B10] Evans RW Applegate WH Briscoe DM Cohen DJ Rorick CC Murphy BT Madsen JC Cost-related immunosuppressive medication nonadherence among kidney transplant recipients Clin J Am Soc Nephrol 2010 5 12 2323 2328 10.2215/CJN.04220510 20847093 PMC2994095

[B11] De Bleser L Matteson M Dobbels F Russell C De Geest S Interventions to improve medication-adherence after transplantation: a systematic review Transpl Int 2009 22 8 780 797 10.1111/j.1432-2277.2009.00881.x 19386076

[B12] Chisholm MA Lance CE Williamson GM Mulloy LL Development and validation of the immunosuppressant therapy adherence instrument (ITAS) Patient Educ Couns 2005 59 1 13 20 10.1016/j.pec.2004.09.003 16198214

[B13] Chisholm-Burns M Pinsky B Parker G Johnson P Arcona S Buzinec P Chakravati P Good M Cooper M Factors related to immunosuppressant medication adherence in renal transplant recipients Clin Transplant 2012 26 5 706 713 10.1111/j.1399-0012.2011.01589.x 22324912

[B14] Chisholm-Burns MA Spivey CA Wilks SE Social support and immunosuppressant therapy adherence among adult renal transplant recipients Clin Transplant 2010 24 3 312 320 19694770 10.1111/j.1399-0012.2009.01060.x

[B15] Chisholm MA Lance CE Williamson GM Mulloy LL Development and validation of an immunosuppressant therapy adherence barrier instrument Nephrol Dial Transplant 2005 20 1 181 188 10.1093/ndt/gfh576 15572384

[B16] Zigmond AS Snaith RP The hospital anxiety and depression scale Acta Psychiatr Scand 1983 67 6 361 370 10.1111/j.1600-0447.1983.tb09716.x 6880820

[B17] Cohen S Hoberman HM Positive events and social supports as buffers of life change stress J Appl Soc Psychol 1983 13 2 99 125 10.1111/j.1559-1816.1983.tb02325.x

[B18] Cohen S Mermelstein R Kamarck T Hoberman HM Sarason IG, Sarason BR Measuring the functional components of social support Social support: Theory, research, and applications 1985 The Hague, Holland: Martinus Nijhoff 73 94

[B19] Cohen S Kamarck T Mermelstein R A global measure of perceived stress J Health Soc Behav 1983 24 4 385 396 10.2307/2136404 6668417

[B20] Cohen S Williamson GM Spacapam S, Oskamp S Perceived stress in a probability sample of the U.S The social psychology of health: Claremont Symposium on Applied Social Psychology 1988 1 Newbury Park, CA: Sage 31 67

[B21] Baker DW Williams MV Parker RM Gazmararian JA Nurss J Development of a brief test to measure functional health literacy Patient Educ Couns 1999 38 1 33 42 10.1016/S0738-3991(98)00116-5 14528569

[B22] Hosmer DW Lemeshow S Applied logistic regression 2000 2 New York: John Wiley & Sons, Inc.

[B23] U.S. Census Bureau Income, poverty, and health insurance coverage in the United States: 2011 2012 U.S. Census Bureau: U.S. Department of Commerce, Economics and Statistics Administration

[B24] Comparison of Medication Adherence Between Once and Twice Daily Tacrolimus in Stable Renal Transplant Recipients [http://clinicaltrials.gov/show/NCT01334333; NLM Identifier: NCT01334333]

[B25] Intervention to Improve Adherence in Teen Kidney Transplant (TAKE-IT) [http://clinicaltrials.gov/show/NCT01356277; NLM Identifier: NCT01356277]

[B26] Medication Adherence in Kidney Transplant Recipients Using Automated Reminders and Provider Notification [http://clinicaltrials.gov/show/NCT01541384; NLM Identifier NCT01541384]

[B27] Nevins TE Thomas W Quantitative patterns of azathioprine adherence after renal transplantation Transplantation 2009 87 5 711 718 10.1097/TP.0b013e318195c3d5 19295316 PMC3580890

[B28] Russell C Conn V Ashbaugh C Madsen R Wakefield M Webb A Coffey D Peace L Taking immunosuppressive medications effectively (TIMELink): a pilot randomized controlled trial in adult kidney transplant recipients Clin Transplant 2011 25 6 864 870 10.1111/j.1399-0012.2010.01358.x 21077956 PMC3130117

[B29] Russell CL Cetingok M Hamburger KQ Owens S Thompson D Hathaway D Winsett RP Conn VS Madsen R Sitler L Medication adherence in older renal transplant recipients Clin Nurs Res 2010 19 2 95 112 10.1177/1054773810362039 20185804

[B30] Schmid-Mohler G Thut MP Wuthrich RP Denhaerynck K De Geest S Non-adherence to immunosuppressive medication in renal transplant recipients within the scope of the Integrative Model of Behavioral Prediction: a cross-sectional study Clin Transplant 2010 24 2 213 222 10.1111/j.1399-0012.2009.01056.x 19674014

[B31] Dharancy S Giral M Tetaz R Fatras M Dubel L Pageaux GP Adherence with immunosuppressive treatment after transplantation: results from the French trial PREDICT Clin Transplant 2012 26 3 E293 299 10.1111/j.1399-0012.2012.01652.x 22686953

[B32] Pai AL Rausch J Tackett A Marsolo K Drotar D Goebel J System for integrated adherence monitoring: real-time non-adherence risk assessment in pediatric kidney transplantation Pediatr Transplant 2012 16 4 329 334 10.1111/j.1399-3046.2012.01657.x 22353189

[B33] Hsiau M Fernandez HE Gjertson D Ettenger RB Tsai EW Monitoring nonadherence and acute rejection with variation in blood immunosuppressant levels in pediatric renal transplantation Transplantation 2011 92 8 918 922 10.1097/TP.0b013e31822dc34f 21857278

[B34] Shemesh E Fine RN Is calculating the standard deviation of tacrolimus blood levels the new gold standard for evaluating non-adherence to medications in transplant recipients? Pediatr Transplant 2010 14 8 940 943 10.1111/j.1399-3046.2010.01396.x 20887400 PMC2992596

[B35] Takemoto SK Pinsky BW Schnitzler MA Lentine KL Willoughby LM Burroughs TE Bunnapradist S A retrospective analysis of immunosuppression compliance, dose reduction and discontinuation in kidney transplant recipients Am J Transplant 2007 7 12 2704 2711 10.1111/j.1600-6143.2007.01966.x 17868065

[B36] Schafer-Keller P Steiger J Bock A Denhaerynck K De Geest S Diagnostic accuracy of measurement methods to assess non-adherence to immunosuppressive drugs in kidney transplant recipients Am J Transplant 2008 8 3 616 626 10.1111/j.1600-6143.2007.02127.x 18294158

